# Supernatants from Newly Isolated *Lacticaseibacillus paracasei* P4 Ameliorate Adipocyte Metabolism in Differentiated 3T3-L1 Cells

**DOI:** 10.3390/biomedicines12122785

**Published:** 2024-12-07

**Authors:** Natalia Grigorova, Zhenya Ivanova, Valeria Petrova, Ekaterina Vachkova, Georgi Beev

**Affiliations:** 1 Department of Pharmacology, Animal Physiology, Biochemistry and Chemistry, Faculty of Veterinary Medicine, Trakia University, 6000 Stara Zagora, Bulgaria; nataliya.grigorova@trakia-uni.bg (N.G.); zhenya.ivanova.12@trakia-uni.bg (Z.I.); valeriya.petrova@trakia-uni.bg (V.P.); ekaterina.vachkova@trakia-uni.bg (E.V.); 2 Department of Biochemistry, Microbiology and Physics, Faculty of Agriculture, Trakia University, 6000 Stara Zagora, Bulgaria

**Keywords:** *Lacticaseibacillus paracasei* P4 and M2.1 strains, postbiotics, adipocyte metabolism, glucose uptake, beta-oxidation, adiponectin

## Abstract

**Background:** *Lacticaseibacillus paracasei* (*L. paracasei*) strains and their postbiotics show potential for managing metabolic disorders such as diabetes and obesity. Two newly isolated *L. paracasei* strains, M2.1 and P4, were yielded from *Formica rufa* anthills in Sinite Kamani National Park, Bulgaria. Their metabolic effects on mature 3T3-L1 adipocytes were investigated. **Methods:** Mature 3T3-L1 adipocytes were treated for 24 h with 10% (*v*/*v*) cell-free supernatants (CFSs) of M2.1 or P4. Two experimental (M2.1, P4) and two control groups (mature, untreated adipocytes and mature adipocytes, treated with 10% (*v*/*v*) MRS broth) were analyzed for intracellular lipid accumulation, glucose uptake, and the mRNA expression of lipid metabolism and beta-oxidation-related genes. Fold changes in gene expression were assessed using RT-qPCR. **Results:** Both M2.1 and P4 CFSs enhanced glucose uptake by over 30% compared to the control. P4 demonstrated a more favorable effect by significantly upregulating adipose triglyceride lipase–patatin-like phospholipase domain containing 2, adiponectin, and peroxisomal beta-oxidation enzymes—acyl-coenzyme A oxidase 1, palmitoyl. Intracellular lipid accumulation increased only with M2.1, while P4 supported improved lipid turnover without promoting excessive lipid storage or lipolysis. **Conclusions:** P4 CFS exhibits the potential to improve adipocyte metabolism by enhancing glucose uptake, promoting beta-oxidation, and increasing adiponectin expression, offering a promising strategy for managing metabolic dysfunctions.

## 1. Introduction

Probiotics are unique microorganisms that confer health benefits to the host when administrated in appropriate amounts. *Lactobacilli*, *Bifidobacteria*, and specific yeast species rank among the microbes with the most robust probiotic characteristics [1,2]. The proper selection and regular consumption of probiotics help balance gut microbiota, enhance gut barrier function, stimulate the immune response, reduce inflammation caused by pathogens, improve digestion, and optimize nutrient absorption [1]. In addition to the well-documented local benefits, there is growing attention to their systemic effects on the host [3,4]. Probiotics are known to improve the lipid profile and insulin sensitivity, reducing inflammation, and the risk of cardiovascular and liver diseases [3,4,5,6,7,8]. These effects are primarily determined by the resorbed metabolic products released during the probiotic’s life cycle, such as short-chain fatty acids, peptides, enzymes, vitamins, and others, collectively known as postbiotics [9,10]. Therefore, a contemporary trend in nutrigenomics is the direct intake of postbiotics, specifically targeted at particular physiological pathways rather than probiotic intake per se [11,12]. The advantages of this approach include greater stability of postbiotics during storage and transport compared to live microorganisms, significantly faster impact, easier and more precise dosing, and strictly specific targeting of a certain health issue, with their effectiveness not dependent on the potential for microbial colonization—a limiting factor for probiotics [9,12,13]. Postbiotics are the preferred choice for people with compromised immune systems or those undergoing severe medical interventions due to the existing risk of bacteremia and other infectious complications [14,15].

Research into the direct effects of postbiotics remains limited. However, evidence suggests that certain probiotic strains exhibit significant antiadipogenic and anti-obesity potential, highlighting their role in modulating body weight and metabolic functions [8,16,17]. These systemic health benefits are mainly based on modulating the physiological state of adipose tissue and the overall level of body inflammation. Besides its well-documented function as an energy storage depot, adipose tissue is a highly metabolic endocrine organ that significantly influences systematic metabolism by producing hundreds of adipokines and biologically active substances [18,19]. Adiponectin, for instance, is known for enhancing insulin sensitivity, inflammation, and the lipid profile and has a significant role in cancer prevention [19,20]. Leptin is well known for regulating appetite and energy homeostasis but also profoundly affects immune system modulations. Thus, the expression levels of adiponectin and leptin influence not only the physiological state of adipocytes but also extend to the overall health of the entire body [19,20]. To improve health outcomes for individuals with obesity, scientists are focusing on dietary supplements that increase the production of beneficial adipokines and enhance beta-oxidation, avoiding the stimulation of lipolysis [21,22,23]. Such targets for developing nutritional strategies extend beyond traditional views of diet and disease, focusing on metabolic modulation at the cellular level for broader systemic health benefits.

It should be noted that different strains, even those closely related, produce distinct, postbiotics and thereby modulate physiological processes in the microorganism in a highly specific manner [24,25,26]. Exploring new sources and strains and deepening our understanding of postbiotic mediators suggests a path for developing dietary supplements for personalized pharmacological strategies that meet the body’s unique needs. There are many beneficial effects described in the literature concerning *Lacticaseibacillus paracasei* (*L. paracasei*) strains [27,28,29]. They comprise antimicrobial, anti-inflammatory, antioxidant, anti-obesity, and lipid metabolism improvement; hypocholesterolemic and stress modulator effects; immune system stimulation; intestinal bacterial microbiota enhancement; and many others.

This study aimed to evaluate the effects of potential postbiotics derived from newly isolated *L. paracasei* strains M2.1 and P4 on adipocyte differentiation, lipid metabolism, and the expression of key genes involved in beta-oxidation. Additionally, the impact on adiponectin, a major health-promoting adipokine, was investigated.

## 2. Materials and Methods

### 2.1. Materials and Chemical Reagents

The current study employed supernatants from newly isolated Bulgarian *L. paracasei* strains M2.1 and P4, as well as 3T3-L1 mouse embryonic fibroblasts (ATCC^®^ CRL-3242™) from the American Type Culture Collection (ATCC, Washington, DC, USA). The reagents used in the current investigation were Dulbecco’s Modified Eagle’s Medium (DMEM) with high glucose content (4500 mg/L), fetal bovine serum (FBS), L-glutamine, an antibiotic solution (Penicillin G, Streptomycin, Amphotericin B), indomethacin, dexamethasone, phosphate-buffered saline (PBS), 100% isopropanol, sodium chloride (HCL), Oil Red O powder, 3-(4,5-dimethyl-2-thiazolyl)-2,5-diphenyl-2*H*-tetrazolium bromide (MTT) powder, a trypsin solution, dimethyl sulfoxide (DMSO), and adipolysis assay kit MAK313—all suitable for cell cultures and purchased from Sigma-Aldrich, Chemie, GmbH (Merk KGaA, Darmstadt, Germany). Insulin (cell application, San Diego, CA, USA) and 3-isobutyl-1-methylxanthine IBMX (Cayman Chemical, Ann Arbor, MI, USA) were also used. The investigated microorganisms were pre-cultured in de Man, Rogosa, and Sharpe (MRS) broth, which was supplied by Oxoid, UK. A Glucose GOD-PAD reagent was purchased from Biolabo SAS (Maizy, France). The plates and pipettes used were sterile and single-use, produced by Corning Incorporated, Costar, Washington, DC, USA. For gene expression analyses, we used the RNeasy Mini Lipid Tissue Kit (QIAGEN Sciences, Inc.; Germantown, MD, USA), the RevertAid First Strand cDNA Synthesis Kit (Thermo Scientific, Waltham, MA, USA), and the KAPA SYBR^®^ fast qPCR Master Mix kit (QIAGEN Sciences, Inc.; Germantown, MD, USA).

### 2.2. Preparation of Microbial Supernatants

*L. paracasei* strains (M2.1 and P4) were isolated from *Formica rufa* anthills in Sinite Kamani National Park, Bulgaria, and identified and cultivated in MRS at 37 °C for 24 h, as previously described [26]. Supernatants from both microorganisms, M2.1 and P4, were filtered through a sterile syringe filter with a pore size of 20 µm (Corning, New York, NY, USA) after centrifugation at 9000 rpm for ten minutes. The obtained supernatants were adjusted to pH 7 with 0.1 N NaOH and then ex tempore included in the freshly prepared adipocyte maintenance culture media (AMM) at a 10% *v*/*v* concentration.

### 2.3. Cultivation and Adipogenesis of 3T3-L1 Cells

3T3-L1 preadipocytes were propagated in basal media (BM) consisting of DMEM, 10% (*v*/*v*) FBS, and 1% antibiotic solution in T75 flasks. They were then seeded in 12- and 24-well plates at 10^4^ cells/mL concentrations and cultured at 37 °C in a humidified atmosphere of 95% air and 5% CO_2_. Upon reaching 100% confluence, the cells underwent a 24 h growth arrest followed by adipogenic differentiation. They were cultured for 48 h in adipogenic induction media (AIM) containing DMEM, 10% (*v*/*v*) FBS, 2% L-glutamine, 0.1 mM IBMX, 0.05 mM indomethacin, 1 µM dexamethasone, 10 µg/mL insulin, and 1% antibiotic solution. In order to achieve full maturation by day 8, the cells were maintained in AMM composed of DMEM, 10% (*v*/*v*) FBS, 2% L-glutamine, 10 µg/mL insulin, and 1% antibiotic solution.

On day 9, the mature adipocytes were divided into two experimental and two control groups. The experimental groups, M2.1 and P4, were treated for 24 h with 10% *v*/*v L. paracasei* M2.1 or P4 CFS in AMM. As a positive control group, mature, untreated cells (IC) were cultured, serving as a baseline for successful adipogenesis. As a Supplementary Material Figure S1 comparison of intracellular neutral lipid accumulation between IC and non-differentiated, untreated (NC) groups at day 8 was provided (Supplementary Materials (Figure S1)). However, an additional control group of mature adipocytes supplemented with 10% (*v*/*v*) (MRS) was included to evaluate the possible metabolic influence of the pure MRS for the same duration.

Three parallel replicates were conducted concurrently for each group (n = 6): Replicate 1 (24-well plates) involved cell viability assessment using the MTT assay; Replicate 2 (12-well plates) included the evaluation of neutral lipid accumulation microscopically and spectrophotometrically by adding Oil Red O staining; and Replicate 3 (12-well plates) was used for measuring glucose and glycerol concentrations in cell supernatants and isolating mRNA from the same 3T3-L1 adipocytes for an RT-PCR gene expression analysis.

### 2.4. Cell Viability Assay

The cell viability of 3T3-L1 upon 24 h treatment with 10% supernatants was determined using the MTT assay. This colorimetric method exploits the reduction in MTT by NAD(P)H-dependent cellular oxidoreductase enzymes, producing an insoluble purple formazan product, as detailed by Yang et al. [30]. In this procedure, cells were incubated with the MTT solution (5 mg/mL) at 37 °C for 80 min. Following incubation, the formazan product was solubilized using an isopropanol solution containing 0.04 N HCl. Absorbance was measured at 570 nm (a reference wavelength of 630 nm) using a Synergy TM Lee Multi-Mode Microplate Reader from BioTek Instruments, Inc., Santa Clara, CA, USA. The results were expressed as a percentage of the control (NC), in line with the approach described by Park et al. [31].

### 2.5. Oil Red O Staining and Intracellular Lipid Accumulation Assessment

At the end of the experiment, differentiated adipocytes were fixed with 10% formalin, dried with 60% isopropanol, and then colored with Oil Red O for 30 min. To quantify neutral lipid accumulation, the stain was extracted from the lipid droplets with 100% isopropanol, and the dye absorbance was measured at 490 nm [32].

### 2.6. Glycerol Concentration Measurement and Lipolysis Rate Estimation

We quantified the glycerol released into the supernatants to evaluate lipolysis in mature adipocytes treated with 10% *L. paracasei* CFS. This analysis used the adipolysis assay kit specifically designed for cell culture supernatants. Each sample was measured twice, at a 570 nm wavelength with a reference correction at 630 nm, to ensure accuracy. Glycerol concentrations were then calculated based on a pre-established concentration curve, following the protocol provided by the manufacturer, and expressed relative to the MRS group as a percent.

### 2.7. Glucose Concentration in Cell Supernatants

After the mature adipocytes were exposed for 24 h to *L. paracasei* M2.1 or P4 CFS, or a 10% solution of MRS broth, we assessed the glucose levels in cell supernatants. This measurement was performed using the Mindrey BS-120 automatic biochemical analyzer manufactured in Guangzhou, China. For the glucose assay, we used the Glucose GOD-PAD reagent. The procedure was carried out according to the manufacturers’ guidelines. To determine the glucose level taken from the treated cells in each group, we subtracted each value (experimental glucose concentration (EG)) from the corresponding amount established in the freshly prepared media just before application (initial glucose concentration (IG)) based on the equation provided by Diaz et al. [33].

Glucose uptake UG (mg/L) = IG − EG

Finally, the data were presented as a percentage of the control group (MRS).

### 2.8. Real-Time PCR

The RNeasy Mini Lipid Tissue Kit was used for total mRNA isolation from pre-lysed mature adipocytes. The quality and quantity of the obtained mRNA were evaluated spectrophotometrically, ensuring that only high-quality mRNA (with absorbance ratios of approximately 2 at 260/280 nm) was used for subsequent analyses. The reverse transcription of equal amounts of mRNA from each sample was performed using the RevertAid First Strand cDNA Synthesis Kit. RT-qPCR was conducted using the KAPA SYBR Green Master Mix, employing self-designed primers as previously described by Grigorova et al. [26]. The RT-PCR data were analyzed using the modified ΔΔCt method, which incorporates normalization based on multiple housekeeping genes. Six housekeeping genes were analyzed: Hypoxanthine Phosphoribosyltransferase (*Hprt*), 18S ribosomal RNA (*18S*), Beta-actin (*Actb*), Ribosomal Protein L19 (Rpl19, also known as 36B4), Glyceraldehyde 3-Phosphate Dehydrogenase (*Gapdh*), and Hydroxymethylbilane Synthase (*Hmbs*). The identification of the least variable gene or combination of genes was performed by the web-based software RefFinder (http://blooge.cn//RefFinder/) accessed on 15 October 2024 [34]. In the final analysis, a combination of *Hprt* and *Actb* was utilized. The sequences of the employed housekeeping and target genes are presented in Table 1.

### 2.9. Statistical Analyses

We utilized Statistica version 10 (StatSoft Inc., 2011, Tulsa, OK, USA) to analyze the obtained data. Initially, descriptive statistics were conducted to calculate the mean and standard error of the mean, which are represented in the figures with values and error bars. The statistical significance of differences was assessed using the non-parametric Mann–Whitney U test.

## 3. Results

### 3.1. Results from MTT Cell Viability Assay

**Figure 1:**
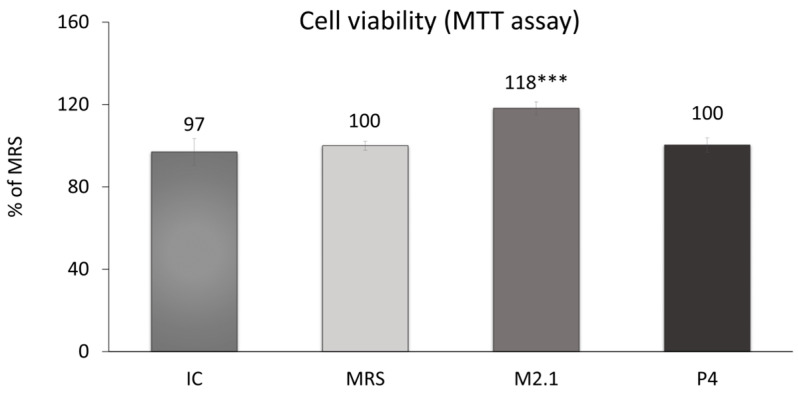
Assessment of cell viability in mature 3T3-L1 cells, treated with 10% cell-free supernatants (CFSs) from *L. paracasei* M2.1 or P4 strains, using the MTT assay: Abbreviations. IC—mature, untreated adipocytes; MRS—mature adipocytes treated with 10% (*v*/*v*) MRS broth (control); M2.1 and P4—experimental groups of mature adipocytes exposed to 10% (*v*/*v*) M2.1 or P4 CFSs. The statistical significance of differences between each experimental group (M2.1 or P4) and the control (MRS) group was evaluated using the non-parametric Mann–Whitney U test. The “asterisk” symbol shows the degree of significance in the figure as follows: *** for *p* < 0.001.

On day 8 following adipogenic induction, mature 3T3-L1 cells were treated with 10% cell-free supernatants (CFSs) from *L. paracasei* M2.1 and P4 for 24 h. Subsequently, an MTT assay was performed to assess any cytotoxic effects of the applied supernatant concentration. As illustrated in Figure 1, no cell-damaging impacts were observed. In fact, cell viability was notably increased by 18% in the samples treated with M2.1 supernatants compared to the control (MRS) (*p* < 0.001).

### 3.2. Intracellular Lipid Accumulation

**Figure 2:**
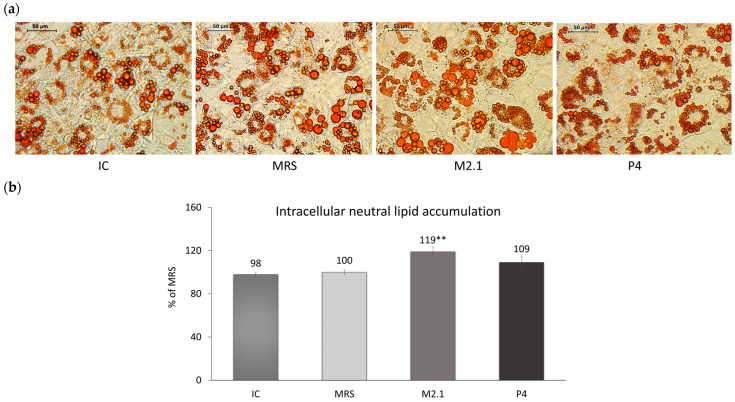
The effect of *L. paracasei* M2.1 and P4 cell-free supernatants (CFSs) on the intracellular neutral lipid accumulation in mature 3T3-L1 cells. (**a**) Microscopic images, stained with Oil Red O (magnification 40×, bars: 50 µm); (**b**) intracellular lipid accumulation after the isopropanol extraction of Oil Red O. Abbreviations: IC—mature, untreated adipocytes; MRS—mature adipocytes treated with 10% (*v*/*v*) MRS broth (control); M2.1 and P4—experimental groups of mature adipocytes exposed to 10% (*v*/*v*) M2.1 or P4 CFSs. The statistical significance of differences between each experimental group (M2.1 or P4) and the control (MRS) group was evaluated using the non-parametric Mann–Whitney U test. The “asterisk” symbol shows the degree of significance in the figure as follows: ** for *p* < 0.01.

Neutral lipid deposition in already differentiated 3T3-L1 adipocytes exposed to 10% cell supernatants from *L. paracasei* M2.1 or P4 was observed microscopically (Figure 2a) and then quantified spectrophotometrically following isopropanol extraction (Figure 2b). Data showed a 19% increase in the intracellular lipid accumulation in adipocytes treated with M2.1 supernatants compared to MRS (*p* < 0.01), with no change observed in the P4 group.

### 3.3. Glucose Uptake and Lipolysis Rate

**Figure 3:**
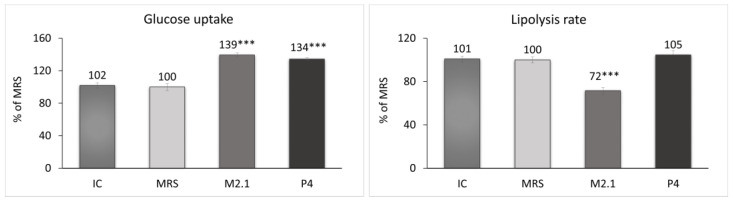
Assessment of the glucose uptake and lipolysis rate after 24 h of treatment of mature 3T3-L1 adipocytes with 10% cell-free supernatants (CFSs) from *L. paracasei* M2.1 or P4 strains. Abbreviations: IC—mature, untreated adipocytes; MRS—mature adipocytes treated with 10% (*v*/*v*) MRS broth (control); M2.1 and P4—experimental groups of mature adipocytes exposed to 10% (*v*/*v*) M2.1 or P4 CFSs. The statistical significance of differences between each experimental group (M2.1 or P4) and the control (MRS) group was evaluated using the non-parametric Mann–Whitney U test. The asterisk symbol shows the degree of significance in the figures as follows: *** for *p* < 0.001.

At the end of the experiment, the glucose concentration and glycerol release in the cell supernatants from all groups were measured. Based on these results, the glucose uptake and lipolysis rate in adipocytes were evaluated (Figure 3). The treated cells demonstrated a significant increase of over 30% in glucose uptake compared to the MRS group (*p* < 0.001). Concurrently, a 28% reduction in the lipolysis rate in the M2.1 group (*p* < 0.001) and no significant change in lipolysis in the P4 group were observed.

### 3.4. Relative Gene Expression

**Figure 4:**
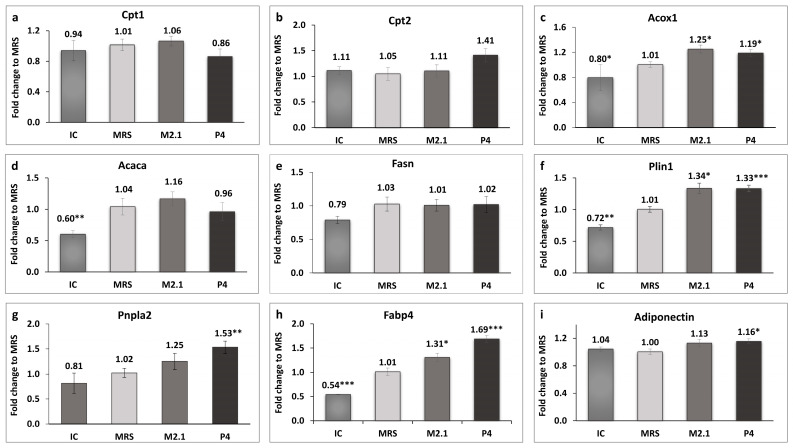
The effect of *L. paracasei* M2.1 and P4 cell-free supernatants (CFSs) on the relative fold change in the gene expression of (**a**) carnitine palmitoyltransferase 1a (*Cpt1*); (**b**) carnitine palmitoyltransferase 2 (*Cpt2*); (**c**) acyl-coenzyme A oxidase 1, palmitoyl (*Acox1*); (**d**) acetyl-CoA carboxylase (*Acaca*); (**e**) fatty acid synthase (*Fasn*); (**f**) perilipin 1 (*Plin1*); (**g**) patatin-like phospholipase domain containing 2 (*Pnpla2*); (**h**) fatty acid-binding protein 4 (*Fabp4*); and (**i**) adiponectin. Abbreviations: IC—mature, untreated adipocytes; MRS—mature adipocytes treated with 10% (*v*/*v*) MRS broth (control); M2.1 and P4—experimental groups of mature adipocytes exposed to 10% (*v*/*v*) M2.1 or P4 cell-free supernatants (CFSs). The statistical significance of differences between each experimental group (M2.1 or P4) and the control (MRS) group was evaluated using the non-parametric Mann–Whitney U test. The “asterisk” symbol shows the degree of significance in the figures as follows: * for *p* < 0.05, ** for *p* < 0.01, and *** for *p* < 0.001.

The expression of genes related to mitochondrial and peroxisomal beta-oxidation in adipocytes was analyzed, as illustrated in Figure 4a–c.

A statistically significant upregulation, exceeding 10%, was observed exclusively in the acyl-coenzyme A oxidase 1, palmitoyl (*Acox1*) gene in both treated adipocyte groups compared to the control (*p* < 0.05). Perilipin 1 (*Plin1*) and fatty acid-binding protein 4 (*Fabp4*) were also significantly upregulated in the M2.1 (*p* < 0.05) and P4 (*p* < 0.001) compared to the MRS group. Interestingly, the patatin-like phospholipase domain containing 2 (*Pnpla2*) and adiponectin (*Adipoq*) showed significantly higher expression only in the P4 group (*p* < 0.01 and *p* < 0.05, respectively).

## 4. Discussion

In Bulgaria, several region-specific microorganisms have been identified for their antiadipogenic, anticancer, and longevity-enhancing properties, such as *Lactobacillus bulgaricus*, *Lactobacillus delbrueckii* subsp. *bulgaricus*, *Lactobacillus helveticus*, *Lactobacillus brevis*, *Lactobacillus plantarum*, etc. [35,36]. Therefore, endemic areas in the country have been studied extensively for probiotic microorganisms with unique health benefits [37,38,39,40,41]. One such area is Sinite Kamani National Park, where we previously obtained four indigenous *L. paracasei* strains (M2.1, C8, C15, and P4) exhibiting significant antidiabetic potential. Among these potentially beneficial strains, we highlighted P4 for its ability to enhance insulin sensitivity and M2.1 for promoting the healthy expansion of mature adipocytes by significantly suppressing their lipolysis. In this context, we recommended a further investigation of their beta-oxidation activity to better understand the mechanism of intracellular action [23].

The results of the present study confirm that 10% CFSs of *L. paracasei* M2.1 and P4, applied in mature 3T3-L1 cells for 24 h, increase glucose uptake by over 30% under simulated high-carbohydrate cell feeding without intensifying lipolysis or exhibiting any noticeable antiadipogenic effects.

The analyzed CFSs appear to regulate the metabolism of mature adipocytes finely, thereby preventing “lipid overload”— a condition associated with sharply reduced adipocyte metabolism, disrupted insulin signaling, induced oxidative stress, and increased inflammatory response [42,43,44]. The data again draw our attention to the possible enhancement of fatty acid beta-oxidation.

Beta-oxidation in mature adipocytes occurs mainly in peroxisomes and mitochondria, with the latter being the primary pathway. This process reduces intracellular lipid accumulation by converting fatty acids into acetyl-CoA and other metabolites. The transport of fatty acids into mitochondria, a critical step in beta-oxidation, is regulated by the enzymes Cpt1 and Cpt2 on the outer and inner mitochondrial membranes, respectively. Cpt1 converts long-chain fatty acyl-CoA into acyl-carnitine, while Cpt2 converts acyl-carnitine back to fatty acyl-CoA in the mitochondrial matrix. The initiation of mitochondrial beta-oxidation is highly dependent on acetyl-CoA carboxylase, which transforms acetyl-CoA to malonyl-CoA, subsequently inhibiting Cpt1 and preventing fatty acid transport into mitochondria [45].

Our study did not establish significant changes in the *Cpt1*, *Cpt2*, and *Acaca* gene expression among the groups, despite the observed trend of increased *Cpt2* gene expression in P4. Therefore, we cannot conclude that the suspected enhanced beta-oxidation occurred in adipocyte mitochondria. Both supernatants, M2.1 and P4, upregulated the expression of the *Acox1* gene, which encodes the first enzyme in the peroxisomal fatty acid beta-oxidation pathway. Its upregulation in adipocytes signifies an increase in the cellular capacity to process fatty acids, thus promoting their utilization for energy, rather than storage [46,47]. Peroxisomal beta-oxidation serves as an additional mechanism for regulating fatty acid balance within the cell, primarily handling medium- and long-chain fatty acids [48,49]. Under certain conditions, such as dietary supplements, fasting, or mitochondrial overload, the peroxisomal beta-oxidation rate can increase significantly, reducing intracellular lipid accumulation in adipocytes. This activation is crucial for maintaining normal metabolic activity and insulin sensitivity in mature adipocytes subject to substantial energy supply [47,50]. The increased breakdown of long-chain fatty acids in peroxisomes carries a risk of intracellular oxidative stress due to the generation of hydrogen peroxide (H_2_O_2_) as a byproduct. However, catalase within peroxisomes rapidly converts H_2_O_2_ into water and oxygen, thereby mitigating the risk of oxidative damage [51].

As mentioned above, peroxisomal beta-oxidation primarily handles medium- and long-chain fatty acids. In our experiment, the source of these fatty acids in both treated groups was questionable. All cells were provided with identical nutrient media, and the groups differed only when the MRS broth in the experimental groups was subjected to microbial fermentation by either M2.1 or P4 for 24 h prior to treatment.

The strains under investigation belong to the *L. paracasei* group [52], known for preferentially fermenting environmental sugars. As a result of their metabolic activity, predominantly short-chain fatty acids, especially lactic acid and acetate, are produced [53,54]. These further influence the expression of specific membrane receptors such as G-protein-coupled receptors 43 and 41, enhancing insulin sensitivity and carbohydrate metabolism rather than beta-oxidation [55]. Nevertheless, they are recognized to affect intracellular fat metabolism positively [45].

Digging deeper, the results outlined the notable discrepancy between the increased gene expression of *Pnpla2* in P4 and the absence of a corresponding rise in lipolysis levels measured in the cell supernatants. Adipose triglyceride lipase (ATGL) (encoded by the *Pnpla2* gene) plays a pivotal role in breaking down triglycerides into free fatty acids, and its upregulation is typically associated with a higher rate of lipolysis [56,57]. Depending on the energy needs, the released fatty acids can also be redirected toward energy production pathways [47]. Our results suggest that these fatty acids probably remain in the cell, serving as substrates for peroxisomal beta-oxidation. Moreover, the upregulation of *Pnpla2* was combined with increased *Plin1* and *Fabp4* gene expressions, which indicate enhanced intracellular lipid mobilization instead of increased lipolysis. The expression of *Plin1* directly influences the accessibility of ATGL to lipid droplets. Plin1 protects lipid droplets from premature or uncontrolled lipolysis and regulates the access of lipases, particularly in adipocytes with increased insulin sensitivity [58,59]. The phosphatidylinositol 3-kinase and cAMP pathway activation reduces protein kinase A activity, decreases Plin1 phosphorylation, and inhibits lipolysis by restricting ATGL access to lipid droplets [60]. However, under certain metabolic conditions, including nutritional stimuli, Plin1 undergoes post-translational modifications such as phosphorylation and facilitates controlled lipolysis, enabling ATGL and other lipases to access and hydrolyze triglycerides [56,61]. This fine regulation of Plin1 activity is part of a dynamic mechanism regulating fat storage and mobilization in adipocytes. The dual function of fat protection and release facilitation is not contradictory, but rather componential within the complex regulation of adipocyte metabolic processes, balancing lipid homeostasis. Therefore, in humans with insulin resistance and obesity, an enhanced, as well as highly suppressed, lipolysis has been reported [62]. The cells treated with P4 and M2.1 CFSs established a notable increase in *Fabp4* gene expression. Fabp4 is a protein with a critical role in the intracellular transport of fatty acids, whose protein expression is often upregulated due to lipolytic stimulation [63]. Its elevated expression enhances the movement of fatty acids, particularly long chains, into various cellular compartments [64]. Thus, in our investigation, the simultaneous upregulation of *Fabp4*, *Pnpla2*, and *Acox1* in white adipocytes from the P4 group suggests that the fatty acids released from intracellular lipid droplets are likely redirected to peroxisomes, where they undergo partial oxidation for energy production.

These interactions contribute to a fine adjustment of the metabolic profile, enhancing the management of the entire cellular machinery within the mature adipocyte. Improved adipocyte metabolism features enhance glucose utilization, established herein, and boost the production of so-called “good adipokines” rather than “bad” ones. Adiponectin is of significant importance to the health status of the entire organism, especially in obesity. It plays a critical role in energy homeostasis, promotes healthy weight maintenance, and prevents obesity-related complications. We established that P4 CFS supplementation to mature adipocytes increases adiponectin gene expression. Adiponectin’s beneficial effects are strongly linked to enhanced fatty acid oxidation, improved lipid metabolism, and increased glucose uptake by adipocytes, all of which are consistent with the findings of this study. Its increased expression is associated with enhanced insulin sensitivity—a fact established in our previous study, where 3T3-L1 cells were treated with the same dose of P4 CFS [26]. Therefore, the upregulation of adiponectin expression along with enhanced beta-oxidation, established in the P4 group, could offer a protective effect against various obesity-related comorbidities.

As a preliminary study investigating potential improvements in mature adipocyte metabolism following supplementation with *L. paracasei* M2.1 and P4 CFSs, it has several limitations. We used gene expression as a proxy for metabolic changes, which may not always reflect functional activity. This approach provides valuable insights but should be further expanded with protein-level validation. Moreover, the obtained results are based on in vitro experiments. Future studies should involve human or animal models to validate our findings and further explore the mechanisms underlying the metabolic improvements observed. Despite these limitations, the current research establishes a strong foundation for future investigations into the metabolic potential of these unique *L. paracasei* strains.

## 5. Conclusions

The study demonstrates that both *L. paracasei* M2.1 and P4 strains increased glucose uptake in mature 3T3-L1 adipocytes without affecting lipolysis or showing antiadipogenic effects. Both strains regulate adipocyte metabolism to prevent “lipid overload”, a condition that disrupts insulin signaling and increases oxidative stress. Notably, P4 CFS upregulated the gene expression of *Acox1*, encoding a key enzyme in peroxisomal beta-oxidation, suggesting enhanced fatty acid processing. This process could reduce lipid accumulation and improve insulin sensitivity. Despite some changes in gene expression, including the upregulation of *Pnpla2*, *Fabp4*, and *Plin1*, lipolysis was not significantly increased, indicating that fatty acids were likely redirected to peroxisomes for partial oxidation. Additionally, P4 CFS increased adiponectin expression, enhancing insulin sensitivity, glucose uptake, and fatty acid oxidation, which could protect against obesity-related complications. These findings highlight the potential of *L. paracasei* strains in improving metabolic health.

## 6. Patents

GB Patent Application No.: 2411546.1.

## Figures and Tables

**Table 1:** Primer Sequences for RT-PCR Analysis.

Abbreviation	Full Name	Forward Primer	Reverse Primer	Product Size (bp)
*Cpt1* NM_013495.2	Carnitine palmitoyltransferase 1a	AAGAACATCGTGAGTGGCGT	GACCTTGACCATAGCCATCCA	165
*Cpt2* NM_009949.2	Carnitine palmitoyltransferase 2	CATCGTACCCACCATGCACT	CTCCTTCCCAATGCCGTTCT	169
*Acaca* NM_133360.3	Acetyl-CoA carboxylase	TGCTCATGTTCCTTGCCCAA	TGCCACCACCATATTTGAGATT	247
*Fasn* NM_007988.3	Fatty acid synthase	CTGAAGCCGAACACCTCTGT	GGGAATGTTACACCTTGCTCCT	218
*Pnpla2* NM_001163689.1	Patatin-like phospholipase domain containing 2	CCTTCACCATCCGCTTGTTG	CCCAGTGAGAGGTTGTTTCG	250
*Plin1* NM_001113471.1	Perilipin 1	ACCCTCCAGAAAAGATCGCC	CTTCCCAGAGCCAGATCAGC	229
*Fabp4* NM_024406.4	Fatty acid-binding protein 4	AACTGGGCGTGGAATTCGAT	CCACCAGCTTGTCACCATCT	150
*Acox1* NM_015729.4	Acyl-coenzyme A oxidase 1, palmitoyl	ACAGAGATGGGTCATGGAACT	ATGTAACCCGTAGCACTCCC	195
*Adipoq* NM_028320.4	Adiponectin	TCCCGTATGATGTGCTTCCT	AGCACAAAACCAAGCAGATGT	157
*Actb* NM_007393.5	β-actin	CCTCTATGCCAACACAGTGC	GTACTCCTGCTTGCTGATCC	211
*Hprt* NM_013556.2	Hypoxanthine guanine phosphoribosyl transferase	ACAGGCCAGACTTTGTTGGA	ACTTGCGCTCATCTTAGGCT	150

## Data Availability

The datasets generated for this study are available from the corresponding author upon request.

## Supplementary Materials

The following supporting information can be downloaded at: https://www.mdpi.com/article/10.3390/biomedicines12122785/s1, Figure S1: Intracellular neutral lipid accumulation in 3T3-L1 cells. (a) Microscopic images, stained with Oil Red O (magnification 40×, bars: 50 µm); (b) intracellular lipid accumulation after isopropanol extraction of Oil Red O.
