# Supplementary material for: Supernatants from Newly Isolated Lacticaseibacillus paracasei P4 Ameliorate Adipocyte Metabolism in Differentiated 3T3-L1 Cells
Source: Biomedicines. 2024 Dec 7;12(12):2785. doi: 10.3390/biomedicines12122785 (PMC11673354; doi:10.3390/biomedicines12122785)
Supplement: Supplementary file 1 [file biomedicines-12-02785-s001.zip › biomedicines-3288930-supplementary.pdf]

a)

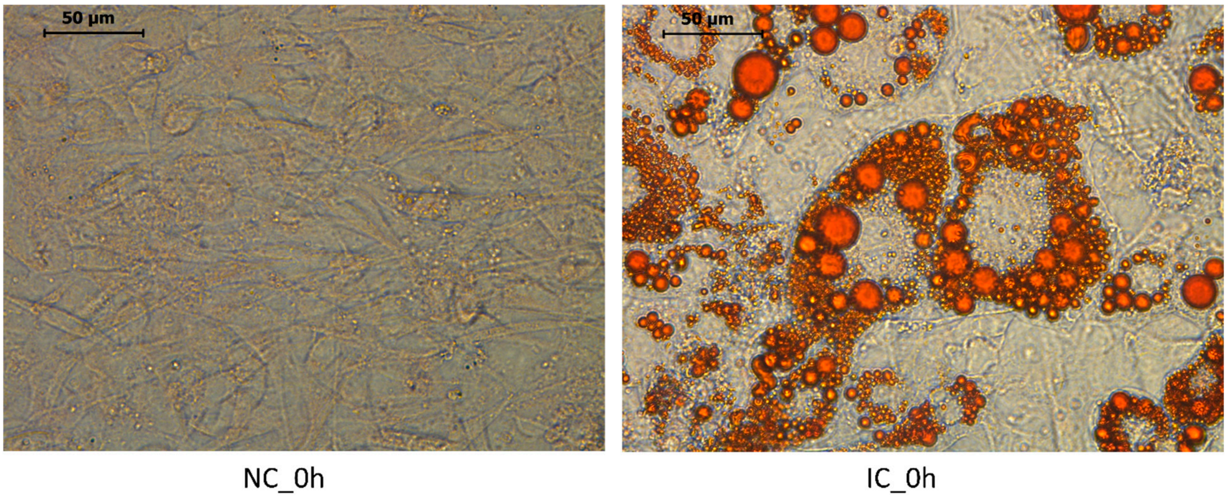

b)

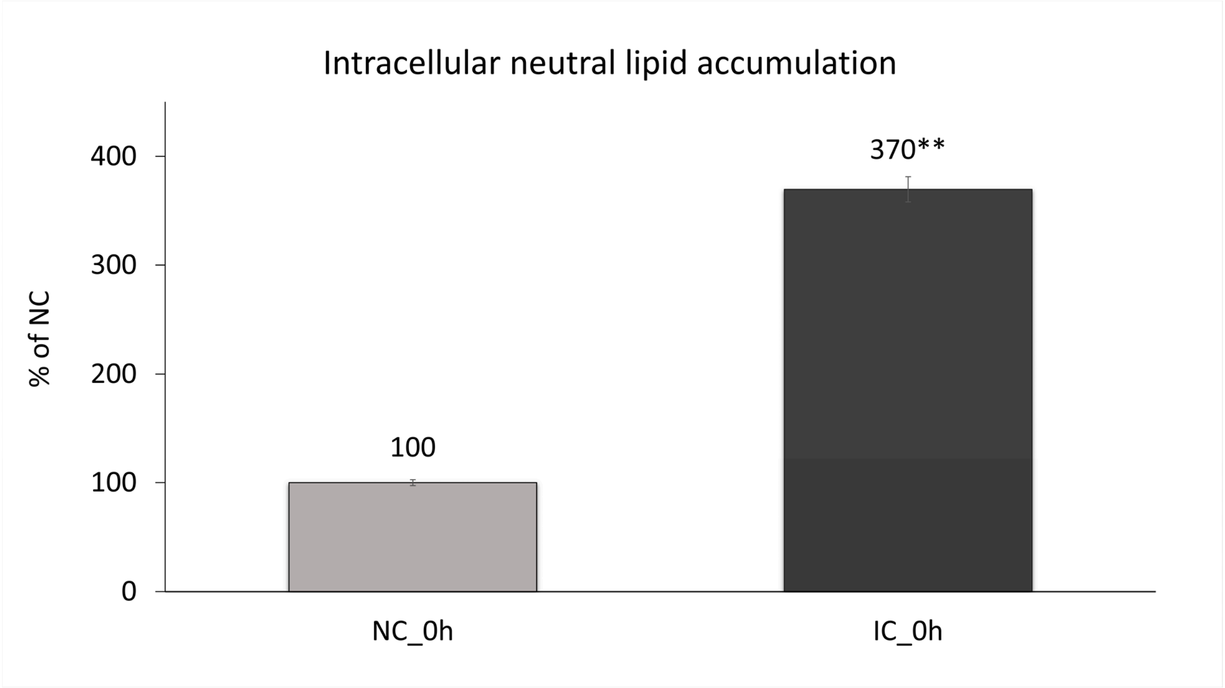

Figure S1. Intracellular neutral lipid accumulation in 3T3-L1 cells at day 8 of adipogenic differentiation. **a)** microscopic images, stained with Oil Red O (magnification 40x, bars: 50 µm); **b)** intracellular lipid accumulation after isopropanol extraction of Oil Red O. **Abbreviations: NC_0h** – non-differentiated, untreated (control); **IC_0h** – mature, untreated adipocytes (control). The statistical significance of differences between groups was evaluated using the non-parametric Mann-Whitney U test. The symbol "asterisk" shows the degree of significance in the figures as follows: ** for $p < 0.01$
